# Assessment of Body Fat Percentage Using B-Mode Ultrasound Technique versus Skinfold Caliper in Obese Healthy Volunteers

**DOI:** 10.7759/cureus.22993

**Published:** 2022-03-09

**Authors:** Avinash S Ingle, Nitin Kumar Kashyap, Soumitra Trivedi, Rajeev Choudhary, Gaurav Suryavanshi, Pugazhenthan Thangaraju, Kiran R Bagale

**Affiliations:** 1 Physiology, All India Institute of Medical Sciences, Raipur, Raipur, IND; 2 Cardiothoracic Surgery, All India Institute of Medical Sciences, Raipur, Raipur, IND; 3 Anatomy, All India Institute of Medical Sciences, Raipur, RAIPUR, IND; 4 School of Studies in Physical Education, Pt. Ravishankar Shukla University, Raipur, IND; 5 Pharmacology and Therapeutics, All India Institute of Medical Sciences, Raipur, Raipur, IND; 6 Biochemistry, Shri Balaji Institute of Medical Science, Raipur, IND

**Keywords:** inference, ultrasound, skin caliper, assessment, fat

## Abstract

Background and aims

The measurement of the skinfold thickness at various sites with the calipers has remained the traditional method for estimation of body fat percentage (%BF) in clinical practice. Although this technique is relatively inexpensive and easy to learn, there are more chances of errors while measuring the skinfold thickness by this method. Therefore, no single standard prediction formula for the determination of body fat could be fixed. The aim of our study was to use B-mode ultrasound (US) for measuring the subcutaneous fat thickness and the calipers for skinfold thickness, and then compare, correlate, and derive the prediction equations for estimation of %BF by both the techniques.

Methods

This cross-sectional, observational, monocentric study was conducted on 43 Indian male volunteers aged 18 to 40 years. After collecting anthropometric data (age, height, weight, body mass index, waist circumference, hip circumference, waist-to-hip ratio [WHR], etc.), the skinfold thickness was measured at four standard sites (biceps, triceps, subscapular region, and suprailiac region) with skinfold caliper (SFC) and then B-mode US. The data were analyzed for distribution, and independent t-test was applied to compare the difference between two means of a %BF estimated by both the methods. The prediction equations were developed from anthropometric and skinfold thickness data obtained from both the methods, i.e., SFC and US, by applying stepwise multiple linear regression.

Results

It was observed that mean values of all the skinfold thicknesses along with the %BF measured by SFC were far more than those measured by US. The %BF measured by US technique (%BF US) was significantly lesser, i.e., 20.69 (SD: 3.126; p < 0.0002), than that of the SFC method (%BF SFC), i.e., 30.38 (SD: 4.634), which is 0.68 % higher. The best prediction equation for the %BF by SFC method was [%BF SFC = -26.154 + 0.208 SFss + 0.374 age + 0.354 SFbi + 32.066 WHR] (R^2 ^= 84.8), where SFss and SFbi are skin fold thicknesses at subscapular and biceps regions, respectively, measured with SFCs, and that by the US method was [%BF US = 0.713 + 0.351 USsi + 0.232 age + 0.248 USss + 0.448 USbi] (R^2 ^= 84.6), where USsi and USss are skinfold measurements at suprailiac and subscapular regions, respectively, measured by US technique.

Conclusion

In our study, we arrived to the conclusion that even though the estimated %BF by both the methods were found to have a significant correlation with each other, the values were very less in case of the US method. In the prediction equations, it was found that the skinfold thickness at the suprailiac region was not found to be the significant determining factor for estimation of %BF by SFC method as that by the US method. Looking at the lesser sample size with all participants being males, we do not recommend the prediction equations to be used in clinical practice in spite of the high R^2^ values.

## Introduction

Lack of physical exercise is well known to contribute to overweight and obesity, which is a risk factor for several noncommunicable diseases (NCDs). In the South-East Asia Region (SEAR), physical inactivity is responsible for 5.1% of deaths, with the incidence of insufficient physical activity ranging from 3% to 41% among males and 6.6% to 64% among females. Bhutan had the highest male and female prevalence (41% and 64%, respectively), followed by the Maldives (37% and 42%, respectively) [1]. According to the recent trial conducted by McKinsey Global Institute, London, 2.1 billion adults of the world are obese (30% of the global population), and obesity is responsible for nearly 5% of all deaths and 20% of the health care expenditure on the prevention and management of obesity [2]. It has been noticed by many researchers that India is also not the exception for it [3,4]. Therefore, the frequent assessment of body composition for the prevention and management of obesity has become a common practice in the outpatient departments (OPDs) of almost all hospitals and clinics.

In view of estimation of various parameters of body composition, techniques that have been under practice include bioelectrical impedance analysis (BIA) for fat-free mass (FFM) and total body water (TBW), use of skinfold thickness for estimation of body fat percentage (%BF), body mass index (BMI), waist-to-hip ratio (WHR), dual-energy X-ray absorptiometry (DeXA), and air displacement plethysmography.

Even though BMI (calculated as weight/height2) is most widely used to assess obesity but as the FFM is also included in weight, the accuracy can be questioned in many conditions (acromegaly, kwashiorkor, Cushing’s syndrome, myxedema, etc.), and waist circumference (WC) and WHR are considered to be superior than BMI in predicting the risk of cardiovascular disease [5]. Previous studies indicated that assessment of %BF by skinfold thickness measurement is easier to be estimated, accurate, reliable, and an important tool for clinicians and fitness professionals, but the major drawback is that inter-observer and intra-observer errors are more while using this technique [6].

Therefore, there is a need of a new method that is accurate, safe, non-invasive, portable, and convenient to measure skinfold thickness. One such technique that has been used by some researchers in the past to measure the thickness of subcutaneous fat is the ultrasound (US) technique.

US is a widely used, non-invasive imaging modality available mainly in radiology, cardiology, medicine, obstetrics & gynecology, anesthesia, trauma-emergency, and now it is being used as a teaching modality for understanding the physiological mechanisms in pre- and para-clinical subjects such as physiology, anatomy, and pathology [7,8]. Therefore, this is our attempt to find out the reliability of the US technique to measure subcutaneous fat thickness in Indian males at standard sites and to compare the results with those by the traditional, i.e., skinfold caliper (SFC) method.

The objective of this study is to estimate and compare the %BF in overweight or obese adults by using US technique to derive the prediction equation for assessment of %BF by this technique in comparison with standard SFC method and to correlate the obtained results with other common obesity parameters such as BMI, WC, and WHR.

## Materials and methods

This study was conducted in the cardiothoracic and vascular surgery OPD of All India Institute of Medical Sciences Raipur, Raipur, India, for a study duration of two years after obtaining the permission from research and ethical committee (550/IEC-AIIMSRPR/2018). The study population of this study included apparently healthy overweight or obese patients visiting the OPD of our institute. The informed consent was obtained, and personal information was noted on predesigned questionnaire performa. The detailed information was provided to all the volunteers before a data collection, which included instructions to be followed, information regarding an instrument, and study procedure. The calculated sample size for this study was 43. This sample size calculation was based on the University of California San Francisco Clinical & Translational Science Institute’s correlational sample calculator.

Sample size was calculated using formula, Sample size = N = [(Zα+Zβ)/C] 2 + 3.

The α (two-tailed) = 0.050. Threshold probability for rejecting the null hypothesis: Type I error rate.

 β = 0.200. Probability of failing to reject the null hypothesis under the alternative hypothesis: Type II error rate.

 r = 0.400 the expected correlation coefficient.

The standard normal deviate for α = Zα = 1.960.

The standard normal deviate for β = Zβ = 0.842, C = 0.5 * ln [(1+r)/(1-r)] = 0.424

So the sample is around 43. The inclusion criteria were all male participants with age ranging from 18 to 40 years and who fulfilled either of the following criteria: BMI > 25 kg/m2, WC > 90 cm, and WHR > 1. Participants with a previous history of any abdominal injury/surgery, and patients with metabolic, endocrinal, cardiovascular, neurological, and kidney diseases were excluded from the study.

Measurement of various parameters

The estimation of BMI was done by measuring height and weight. Height was measured by using a wall-mounted, non-extensible measuring tape with the participant in the standing position with feet together. The body weight was measured by a digital weighing machine with high precision mechanism with participants wearing light clothes and no shoes and with empty stomach. The BMI was calculated according to the Quetelet index: BMI = Weight (in kg) / height (in meters^2^). The BMI range of 20 to 24 kg/m^2^ was considered as normal, BMI 25-29 kg/m^2^ as overweight, and BMI > 30 kg/m^2^ as obese [9]. WC (normal < 90 cm for Asian males) was measured with the participant in erect and relaxed standing posture with feet together to the nearest mm midway between the subcostal and transtubercular plane with a non-extensible measuring tape at the end of a normal expiration. Two measurements with difference not more than 1 cm were averaged. Participants with WC more than or equal to 90 cm were considered as overweight. The WHR is calculated based on hip and WC. The hip circumference (HC) was measured at the gluteal region of maximum protrusion with tape parallel to the ground. The participant was made to relax in the standing posture with hands spreading wide. Two measurements with a difference of not more than 1 cm were averaged. males with WHR of > 1.0 were considered to be overweight or obese [5,10].

Estimation of %BF by using SFC

It is an indicator of excess overall subcutaneous fats which was calculated by taking skinfold thickness at various standard sites on dominant side by using skin caliper (Harpender, HSB-BI, Baty International, West Sussex, UK) in the erect and standing position, with arms relaxed [6].Two readings were taken at each skinfold of following sites and then averaged. Site 1 (biceps) corresponds to the anterior of right biceps midway between the axilla and antecubital fossa, site 2 (triceps) corresponds to the midline over the triceps muscle between the tips of acromion and olecranon processes, site 3 (subscapular) corresponds to the skinfold at the inferior angle of the scapula, and site 4 (suprailiac) corresponds to the diagonal fold above the crest of ileum in the midaxillary line. The %BF was calculated from standard Durnin and Womersley regression equation by using the values of skinfold thickness obtained by SFC [6].

Estimation of body fat percentage by using US (%BF US):

The subcutaneous fat thickness was measured at mentioned sites with standard US technique by using a US machine (GE, Raipur, Chhattisgarh) in B-mode with a high-frequency transducer of 5 MHz to achieve better resolution with lesser penetration [11]. To avoid skin compression, sufficient amount of jelly was used so that the images were acquired with minimal pressure on the skin [9]. Similarly, the percentage body fat using US (%BF US) was calculated from standard Durnin and Womersley regression equation (as like by SFC method) by using the values of subcutaneous fat thickness obtained by US [7,12].

Statistical analysis

Data were analyzed for distribution, and independent t-test and stepwise multiple linear regression analysis were applied.

 A p-value of <0.05 was considered statistically significant. After comparing the %BF values, estimated by SFC and US methods with independent t-test, we found that the results obtained were significantly different (t= 0.03). Table 1 shows the mean values of all the demographic parameters along with those of all the skinfold thickness and subcutaneous fat thickness. It is observed that mean values of all the skinfold thicknesses measured by SFC were far more than those of subcutaneous fat thicknesses (measured by US technique), affecting the %BF calculated using the Durnin and Womersley equation as well. It was noted that %BF calculated from subcutaneous fat thickness measured by US technique (%BF US) was significantly lesser, i.e., 20.69 (SD: 3.126; p < 0.0002) than measured using the SFC method (%BF SFC), i.e., 30.38 (SD: 4.634), which is 0.68 % higher than that of US method (%BF US).

**Table 1:** Descriptive statistics SFT, skinfold caliper thickness

	Mean	Standard Deviation
Age (years)	35.07	6.54
Weight (kg)	79.72	8.41
Height (cm)	1.69	.07
Body mass index (kg/m^2^)	28.03	2.09
Waist circumference (cm)	102.02	5.73
Hip circumference (cm)	98.86	5.12
Waist-to-hip ratio	1.03	.02
SFT by skinfold caliper: biceps (mm)	7.85	2.19
SFT by ultrasound method: biceps (mm)	5.07	1.87
SFT by skinfold caliper: triceps (mm)	18.74	5.37
SFT by ultrasound method: triceps (mm)	9.72	2.73
SFT by skinfold caliper: subscapular (mm)	33.18	10.22
SFT by ultrasound method: subscapular (mm)	13.66	4.95
SFT by skinfold caliper: suprailiac (mm)	40.73	13.98
SFT by ultrasound method: suprailiac (mm)	16.93	4.94
Body fat % by skinfold caliper	30.39	4.63
Body fat % by ultrasound method	20.69	3.13

The prediction equations were derived by applying multiple regression analysis (stepwise) method, which makes the model fit for estimating %BF SFC and %BF US considering them as dependent factors. The Durbin-Watson score was calculated to 2.021 as this is in the range of 0-4 which proves the validity of the test applied.

## Results

Assumptions

To test whether the assumptions are fulfilled or not, we studied the residual statistics, normal P-P plot, and scatter plot of regression standardized residuals. The residual statistics in Table 2 shows that all the standard residuals lay between -3 and +3, while dependent variable is %BF SFC.

**Table 2:** Residuals statistics Dependent variable: % BF SFC %BF, body fat percentage; SFC, skinfold caliper

	Minimum	Maximum	Mean	Standard Deviation	N
Predicted value	21.16	44.54	30.39	4.34	43
Residual	-3.63	2.57	0.00	1.62	43
Standard predicted value	-2.13	3.26	0.00	1.00	43
Standard residual	-2.11	1.49	0.00	0.94	43

**Figure 1:**
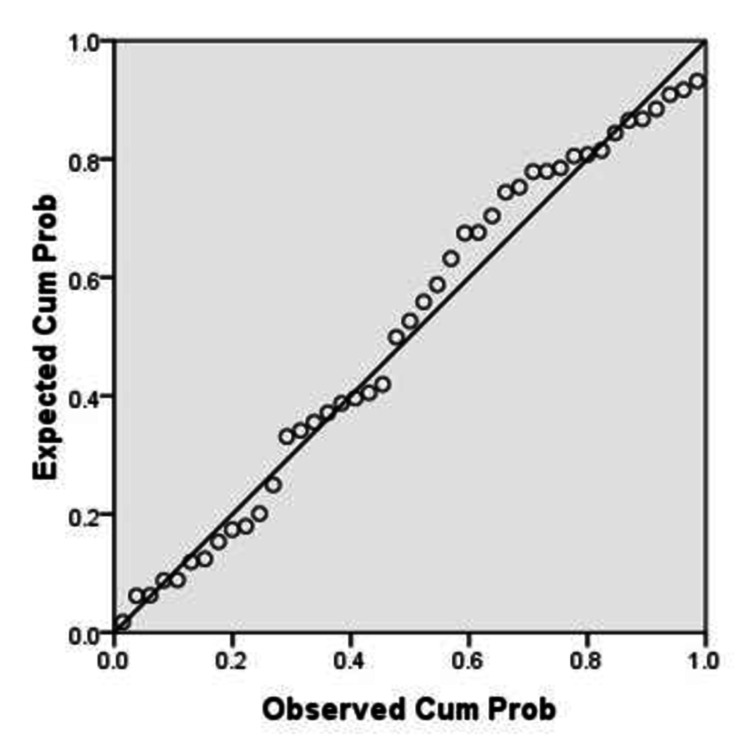
P-P plot Dependent variable: %BF SFC %BF, body fat percentage; SFC, skinfold caliper

**Figure 2:**
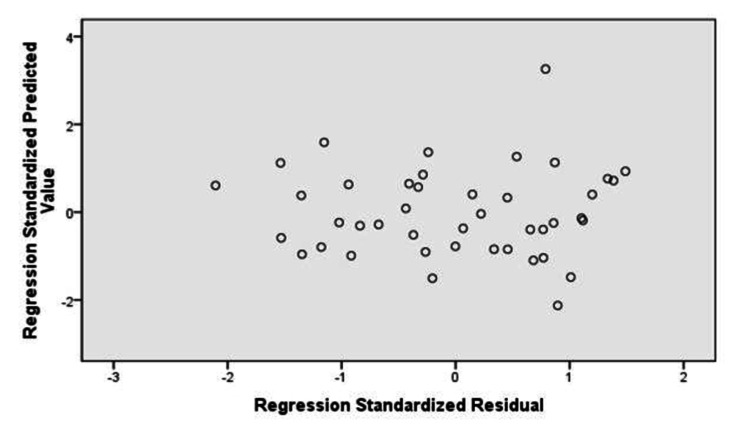
Scatter plot Dependent variable: %BF SFC %BF, body fat percentage; SFC, skinfold caliper

Normal P-P plot (Figure 1) and scatter plot (Figure 2) of regression standardized residuals for %BF by SFC (%BF SFC) as a dependent variable shows that they were normally distributed and constant variance in residuals was found.

The residual statistics in Table 3 shows that all the standard residuals lay between -3 and +3, while the dependent variable is %BF US.

**Table 3:** Residuals statistics when the dependent variable is %BF US %BF, body fat percentage; US, ultrasound

	Minimum	Maximum	Mean	Standard Deviation	N
Predicted value	11.10	23.32	16.58	3.19	43
Residual	-2.97	3.79	0.00	1.48	43
Standard predicted value	-1.72	2.12	0.00	1.00	43
standard residual	-1.85	2.37	0.00	0.93	43

**Figure 3:**
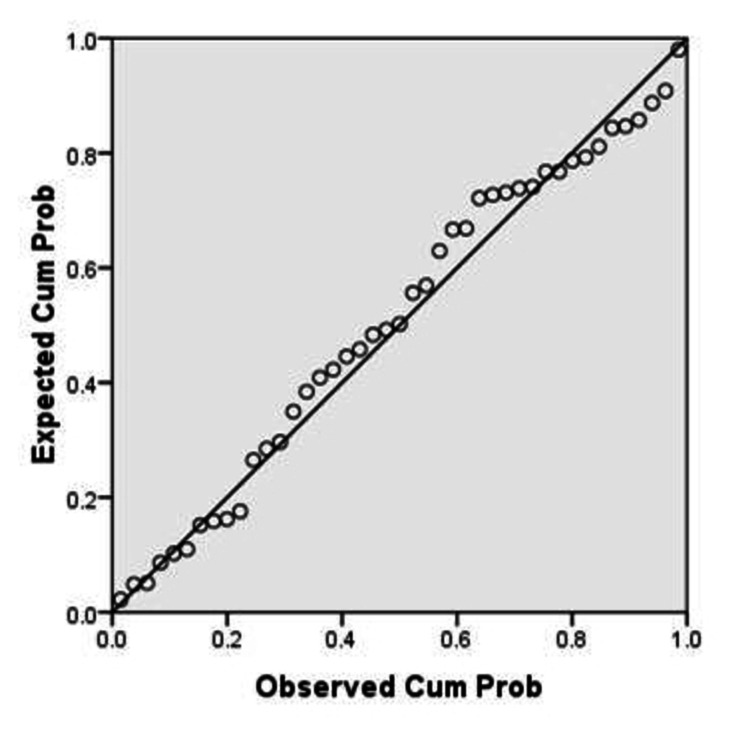
P-P plot Dependent variable: %BF US %BF, body fat percentage; US, ultrasound

**Figure 4:**
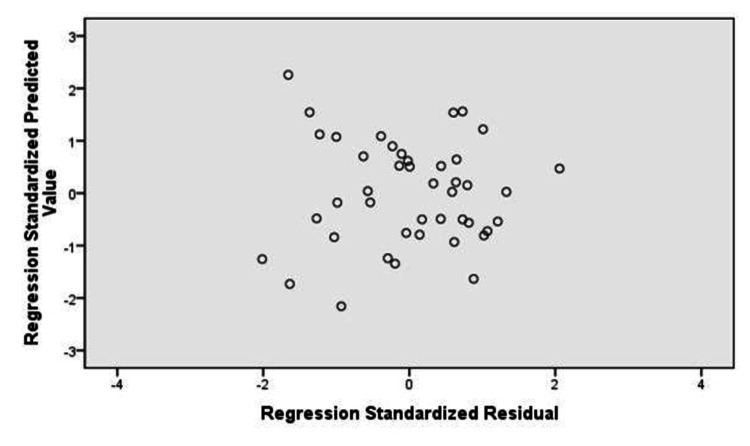
Scatter plot Dependent variable %BF US %BF, body fat percentage; US, ultrasound

The P-P plot (Figure 3) and scatter plot (Figure 4) of regression standardized residuals for %BF by using US method (%BF US) as a dependent variable shows that they also were normally distributed and constant. Thus, all three normality assumptions were fulfilled.

From Table 4 showing the model summary of skinfold method, it was estimated that 52.6% of the %BF using SFC technique was determined by subscapular skinfold thickness alone, whereas just by adding factors such as age, skinfold thickness at biceps region, and WHR, 84.8 % of the %BF SFC was explained. The %BF SFC = 18.896 + 0.334 SFss [adjusted R^2^ = 52.6], %BF SFC = 6.879 + 0.219 SFss + 0.368 age + 0.342 SFbi [adjusted R^2^ = 82.9], and %BF SFC = -26.154 + 0.208 SFss + 0.374 age + 0.354 SFbi +32.066 WHR [adjusted R^2^ = 84.8], where SFss and SFbi are skinfold thicknesses at subscapular and biceps regions, respectively, measured with SFCs. The best possible models for estimating %BFSFC and %BF US were established by analysis of variance (ANOVA).

**Table 4:** Model summary: skin fold method Model 1 predictors (constant): SFss Model 2 predictors (constant): SFss and age Model 2 predictors (constant): SFss, age, and SFb Model 3 predictors (constant): SKss, age, SKb, and WHR Dependent variable: percent body fat by skinfold caliper All other measured variables were excluded because of an insignificant contribution SFss, skinfold thickness at subscapular region measured by skinfold caliper; SFb, skinfold thickness at biceps by skinfold caliper

Model	R	R^2^	Adjusted R^2^	Standard Error of the Estimate	Durbin-Watson
1	0.73	0.53	0.51	3.23	
2	0.89	0.79	0.78	2.18	
3	0.92	0.84	0.83	1.92	2.02

While in case of estimation of %BF by US method, as given in Table 5, which shows the model summary, the suprailiac skin thickness was responsible for 49.3%, whereas by adding age, subscapular skin thickness, and biceps skin thickness measured by US techniques, 84.6 % of the %BF was estimated with the following predicted equations, i.e., %BF US = 12.780 + 0.486 USsi [adjusted R^2^ = 49.3], %BF US = 1.416 + 0.436 USsi + 0.232 age + 0.272 USss [adjusted R^2^ = 77.7], and %BF US = 0.713+ 0.351 USsi + 0.232 age + 0.248 USss + 0.448 USbi [adjusted R^2^ = 84.6], where USsi and USss are skinfold measurements at suprailiac and subscapular regions, respectively, measured by US technique.

**Table 5:** Model summary: ultrasound technique Model 1 predictors (constant): USsi Model 2 predictors (constant): SSsi and age Model 2 predictors (constant): SSsi, age, and USss) Model 3 predictors (constant): SSsi, age, USss, and USbi Dependent variable: percent body fat by US method All other measured variables were excluded because of an insignificant contribution. USsi, skin thickness at suprailiac region measured by US method; USss, skinfold thickness at biceps measured by US method

Model	R	R^2^	Adjusted R^2^	Standard Error of the Estimate	Durbin-Watson
1	0.70	0.49	0.48	2.25	
2	0.83	0.69	0.67	1.79	
3	0.89	0.79	0.78	1.48	2.20

While carrying out correlation by applying Pearson correlation method, it was observed that the skin thickness values measured by both the methods at all the aforementioned sites were significantly correlating with each other. We also observed that the values of %BF by SFC method were significantly correlating with age, WC, and HC, but not with other factors such as age, weight, height, BMI, and WHR.

The values of %BF by US method were significantly correlated with weight and height but didn’t show any significance with BMI, WC, HC, and WHR.

## Discussion

Overweight and obesity, which are risk factors for several NCDs, are exacerbated by a lack of physical activity; 150 minutes of moderate to vigorous physical exercise each week is expected to reduce the risk of ischemic heart disease by 30%, diabetes by 27%, and breast and colon cancer by 21%-25% [1]. The standard method for measuring the total %BF is DEXA but since it is difficult to perform, time-consuming, and expensive, and involves the radiations, we chose the traditional and most common one such as the SFC method to compare with US. It is the most commonly used method for measuring the %BF in which again there are four site, seven site, and eight site methods. The most frequently used method is four sites one, i.e., biceps, triceps, subscapular region, and suprailiac region. It was developed by Durnin and Womersley to calculate the %BF. Earlier studies have reported under- and overestimation of the %BF by this technique, which might be because the fat and dermis thickness are measured twice while measuring the skinfold thickness. It is well known that there are variations are possible while holding the skinfold from individual to individual and depend on laxity and elasticity of the skin. That is the reason why there is a need of new, easy, and accurate method for the estimation of %BF, lean body mass, body fat mass, etc. Therefore, we tried to use very commonly available, portable, accurate, and harmless technique of US for the measurement of %BF in comparison to the SFC method [13,14].

In this study, although there were significant correlations between the measurements of corresponding skinfold thicknesses including the %BF by both the methods, the SFC values were observed to be more than those measured by the US method. Similarly, Carla Perez-Chririnos Buxade et al and Wagner also observed same for all sites except for biceps and abdominal sites while assessing the intra-rater and test-retest reliability of skinfold and A-mode US methods [11,15]. On the contrary, Booth et al. in 1966 observed that the average caliper readings were almost 60% of the US readings for all measured sites, which might be because of the fact that the calipers are known to cause compression of the fats and the study did not mention about the caliper pressure on the skin [16].

In our study, the variables such as subscapular and biceps skinfolds, and age were determining 82.9% of %BF with the SFC method, whereas the 84.6% of the same was determined by suprailiac, subscapular, and biceps skinfold thickness values with a US method.. But in the contrary, Nosslinger et al. also noted that although the results by both methods were correlating with each other, the best agreement of the fat percentage was obtained from thigh fold and subscapular fold [17]. Neves et al. predicted the equation for US method by measuring skinfold thicknesses at nine sites, and factors such as triceps, thigh, and subscapular skinfolds along with age were determining only the 60% of body fat [18].

In our prediction equations, the skinfold thickness at the suprailiac region which, is the most common and prominent site of fat accumulation, was not found to be the significant determining factor for estimation of %BF by SFC method as that by the US method. This might be the result of the variations in skinfold holding and the inability of SFC to differentiate subcutaneous fat layer from dermis and also from the underlying muscles, especially at the compressible sites such as the suprailiac region.

But as per the prediction formula of Thiebaud et al., anterior abdomen, triceps, and lower limb sites were the main determinants of the %BF [19]. While comparing the mean values of the measurements obtained from both methods, we noted the significant difference (p<0.0002, unpaired t-test) between two means for all the measurements. This might be because we have excluded the thickness of epidermis, dermis, and fascia while measuring the subcutaneous adipose tissue thickness, whereas in skinfold measurement by caliper technique that is also measured. As we know that the thickness of the skin also varies individual to individual and site to site of the body parts, we excluded the thickness of skin, superficial, and deep fascia, which was possible only with US technique only while conducting this study.

In our study, it was noted that BMI did not come out to be a determinant of adiposity, which was also observed by previous studies as well [20]. This may be because the muscle mass and lean body mass are also measured while calculating the BMI.

Limitations of the study

The main limitation of our study was that all of our participants were overweight males and therefore the trends in females could not be ascertained. The reason behind including male participants is technical difficulty raised during screening with female participants. India being a developing setup, female participants refused the measurement being done at body sites. As we used the Durnin and Womersley regression equation for the estimation of %BF by using four sites, the results would have been more précised if seven site measurements would have been used. We could not achieve the calculated sample size (four short of the calculated number) because of sudden lockdown during the COVID-19 pandemic. We have only focused on the dominant side of the participants as few of them provided only short duration for conduct of the study. Looking at the lesser sample size with all participants being males, we do not recommend the prediction equations to be used in clinical practice in spite of the high R^2^ values.

Implications

The results obtained from this study shall be used by the clinicians to calculate the adiposity along with the risk of developing the NCDs and other obesity-related diseases much accurately by using this common bedside, less time-consuming, less cumbersome, and cost-effective method of determining body fat composition [21].

## Conclusions

All the skinfold measurements along with the %BF are found to be more for traditional SFC method than the US method. This might be the result of the variations in skinfold holding and the inability to differentiate subcutaneous fat layer from dermis and sometimes underlying muscles, particularly at the compressible sites such as the suprailiac region. This might be the reason why the skinfold thickness at the suprailiac region, which is the most common and prominent site of fat accumulation, was not found to be the significant determining factor for estimation of %BF by SFC method as that by the US method.
